# Effectiveness of an intervention program to develop e-cigarette control leaders at the University in Lampang Province, Thailand

**DOI:** 10.18332/tpc/192694

**Published:** 2024-09-18

**Authors:** Supa Vittaporn, Krailat Kanthajaem, Arpapon Coothongkul, Kasama Pooseesod

**Affiliations:** 1 Faculty of Public Health, Thammasat University, Lampang, Thailand; 2 Thammasat University Research Unit in Environment, Health and Epidemiology, Thammasat University, Pathum Thani, Thailand; 3 Student Affairs Division, Thammasat University, Lampang, Thailand; 4 Health Promotion Unit, Thammasat University Hospital, Pathum Thani, Thailand; 5 Thammasat University Research Unit in One Health and Ecohealth, Thammasat University, Pathum Thani, Thailand

**Keywords:** e-cigarettes, student leader, undergraduate student, participatory action research, Thailand

## Abstract

**INTRODUCTION:**

The use of e-cigarettes is increasing worldwide, especially among young adults. Due to the health risks, this study aimed to assess undergraduate students' e-cigarette use and attitudes toward them, and evaluate the effectiveness of an intervention program to develop e-cigarette control leaders at the University in Lampang province, Thailand.

**METHODS:**

Participatory action research (PAR) was conducted among 46 undergraduate students. To assess the situations of undergraduate students' e-cigarette use and attitudes toward them, in-depth interviews were conducted with 18 of those students – nine users and nine non-users. The remaining 28 were student leaders who were given questionnaires and took part in focus groups to evaluate the effectiveness of the intervention program in developing e-cigarette control leaders. Descriptive statistics and the Wilcoxon signed rank test were used to analyze quantitative data. The qualitative data were analyzed using a thematic analysis of the content. This study took place at the University in Lampang province, Thailand, in 2023.

**RESULTS:**

Regarding the use of e-cigarettes on the part of undergraduate students and their attitudes about their use, the majority of users stated that e-cigarettes were accessible, appealing, and more socially acceptable than conventional cigarettes. However, most non-users cited vapor smell and health impacts as their main reason for not using e-cigarettes. The intervention program to develop leaders in e-cigarette control could significantly enhance the leaders' knowledge (p<0.001) and attitude regarding e-cigarettes (p=0.001). After their anti-e-cigarette campaign, the soft skills and managerial abilities of the leaders in e-cigarette control improved, and the knowledge and attitude regarding e-cigarettes of undergraduate students who attended the campaign also increased.

**CONCLUSIONS:**

The intervention program to develop leaders in e-cigarette control resulted in positive outcomes. This program could enhance the leaders' knowledge and attitude regarding e-cigarettes. Their soft skills and managerial abilities in e-cigarette control also improved.

## INTRODUCTION

The use of e-cigarettes has increased worldwide^1^. In 2021, there were 82 million vapers globally and 14.3 million vapers in South-East Asia^2^. Among young adults, the use of e-cigarette sites rose dramatically^3^. The factors behind the rise in e-cigarettes among them is complex, but the most frequent causes of e-cigarette use have been identified as: curiosity, having a friend or family member who uses them, the availability of flavors, and the belief that e-cigarettes are a safe and effective option to quit smoking^4-6^.

E-cigarettes are a form of addictive substance that can act as a gateway to other addictive drugs^7^. The aerosols from e-cigarettes include substances that are harmful to the body. These substances include small particles that penetrate deeply into the lungs, as well as cancer-causing chemical substances^8^.

Thirty-four countries banned the sale of e-cigarettes, 88 did not set a minimum age for purchasing e-cigarettes or cigarettes, or implemented any laws governing these dangerous products^9^. Although e-cigarettes are prohibited in Thailand, young adults, particularly undergraduate students, have been using e-cigarettes at a significantly higher rate in the past few years^10,11^. The e-cigarette use among the Thai students has been associated with several factors, including knowing others who use e-cigarettes, having a neutral attitude toward e-cigarette use, perceiving e-cigarettes to be less dangerous, and believing that using e-cigarettes in public is not illegal^10-12^. Thus, Universities should design a measure to prevent and control the use of e-cigarettes among undergraduate students by developing an intervention program.

Youth participatory action research (PAR) has had favorable health outcomes^13^. The identified consequences of PAR for youth, include knowledge acquisition, social justice perception, social cognitive development, interactions with adults, community connection and youth, and empowerment as change agents. Numerous PAR studies involving youths have demonstrated successful tobacco prevention outcomes^14,15^. The PAR method could assist youths in creating an action plan that includes holding strategic meetings and workshops to increase critical awareness among peers^14^. Although there are numerous studies using PAR with youth in the context of Thailand^16-20^, no study has thus far used the PAR method to develop and evaluate an intervention program to develop e-cigarette control leaders in the university setting. Undergraduate students are suitable candidates to lead e-cigarette prevention initiatives in universities through PAR because student leaders can influence other peers’ perceptions of cigarette products^21^. Thus, the present study used the PAR approach to: 1) diagnose the situations of undergraduate students’ e-cigarette use and attitudes toward them, 2) use the information of this situation in planning an intervention program aimed at generating e-cigarette control leaders, 3) conduct the intervention program including indoor and outdoor training activities, and 4) evaluate the effectiveness of the intervention program. This study hypothesized that the intervention program could develop e-cigarette control leaders at the University in Lampang province, Thailand.

## METHODS

### Design and setting

This is the quasi-experimental study, which took place at the suburban University in Lampang province, Thailand, from March to July 2023. This university has a campus located about 15 km away from Lampang city.

### Participants and recruitment

Participants in this study were undergraduate students of the University, Lampang province, Thailand. The criteria of the participants were that they be undergraduate students (aged ≥18 years) who could communicate in the Thai language, were willing to take part in the study and willing to sign an informed consent form before the study started.

This study was conducted among 46 undergraduate students. Eighteen students were selected to participate in in-depth interviews regarding the situations of undergraduate students’ e-cigarette use and attitudes toward them. The potential participants were selected through snowball sampling. Nine were current or former e-cigarette users, and nine were non-users. The other 28 were student leaders who received questionnaires and participated in focus groups to evaluate the effectiveness of the intervention program in developing e-cigarette control leaders. These leaders represented student committees and student clubs. The G*Power software version 3.1.9.4^22^ was utilized to determine the sample size of student leaders (n=28) for the intervention. A t-test was used to calculate the alpha error level, which was 0.05. The power of the test was set at 0.85, and the expected effect size was 0.59^23^. Using a lottery, researchers selected potential participants at random from lists of 35 student leaders who applied for the training program.

## Procedure

PAR was used in this study. The PAR process cycle in this study consisted of the stages of problem identification, planning, intervention, and evaluation^24^. Both qualitative and quantitative methods were utilized to collect and evaluate data. The details pertaining to the four study stages are as follows.

*Stage 1 – Diagnosing*

Meetings with the research team and stakeholders, including two university student-affairs practitioners, one student-council president, two nurses, and one police personnel involved in the e-cigarette problem, were the first steps in explaining the objectives of the study and getting prepared to identify the e-cigarette problem. Over the course of two weeks, the situations of undergraduate students’ e-cigarette use regarding the accessibility, vaping behavior, and incentivizing and disincentivizing features of e-cigarettes, as well as attitudes about e-cigarette use, were assessed through in-depth interviews with 18 undergraduate students. These qualitative data were analyzed by the research team for use in building an intervention program aimed at generating e-cigarette control leaders among volunteer undergraduate student leaders.

*Stage 2 – Planning action*

Stakeholders participated in two participatory meetings to develop an intervention program for developing e-cigarette control leaders based on the above qualitative findings. This program aimed to improve the leader’s knowledge and attitude regarding e-cigarettes and enhance their communication skills. Three experts established content validation of the intervention program before implementation.

*Stage 3 – Taking action*

The program included training (indoor) and outbound (outdoor) activities. The indoor training comprised boosting their knowledge and attitude regarding e-cigarettes and enhancing their communication skills by: 1) teaching about the hazards of e-cigarettes and the laws that regulate them, in the form of PowerPoint presentations; 2) establishing group discussions about e-cigarette use; and 3) practicing communication about risk information to e-cigarettes users. This training took 8 hours/day for three days and was conducted by a tobacco treatment specialist and law expert. After indoor training, the leaders were requested to plan and conduct a four-month campus-wide anti-e-cigarette campaign as part of their outdoor training. They were separated into three small groups and assigned to create a campaign aimed at deterring undergraduate students from starting e-cigarettes and encouraging them to quit as soon as possible. They received some budget for the campaign development (US$225 per group). Their plan was adjusted with three experts before launching their campaign. The campaign activities which were organized and run by the leaders included: 1) a one-day exhibition, ‘Be smart, don’t start vaping’; 2) three-hour sessions of lecture and seminars; 3) online e-cigarette-quiz competitions; 4) e-posters with information about advice on quitting cigarettes and prevention of vaping; 5) one anti-vaping short film; and 6) three printed posters of anti-vaping messages.

*Stage 4 – Evaluating action*

**Figure 1:**
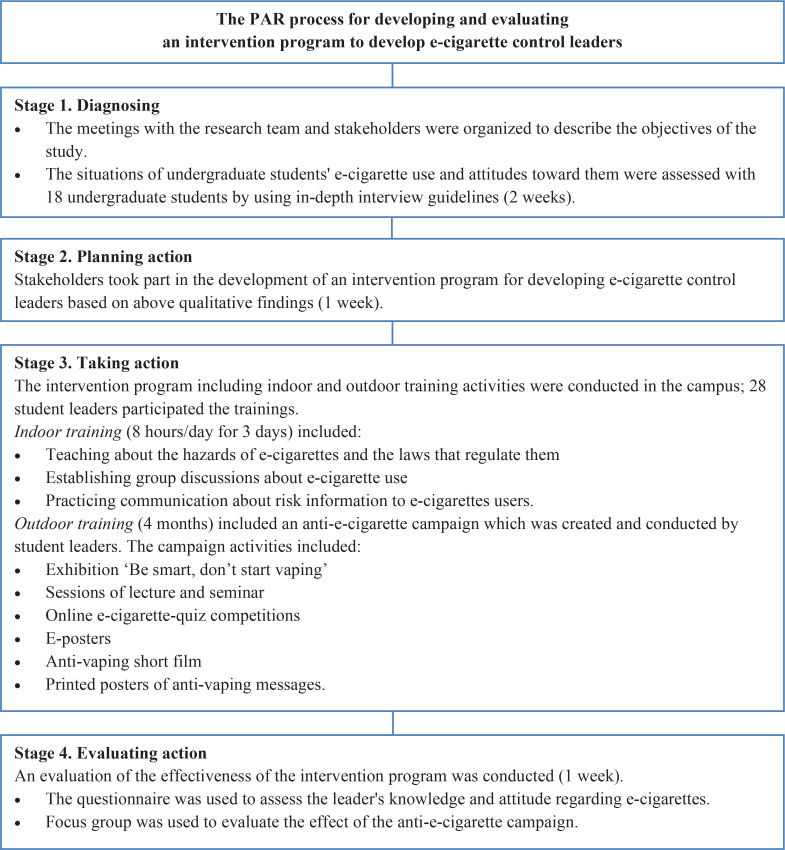
Flow diagram of the PAR process for developing and evaluating an intervention program to develop e-cigarette control leaders at the University in Lampang Province, Thailand

At this stage, an evaluation of the effectiveness of the intervention program in developing e-cigarette control leaders was carried out. Individual-level changes in the leaders’ knowledge and attitudes regarding e-cigarettes were assessed using research questionnaires. An evaluation and assessment of group-level changes by examining the effect of the anti-e-cigarette campaign were carried out through a focus group discussion. The flow diagram of the PAR process for developing and evaluating an intervention program to develop e-cigarette control leaders in this study is shown in Figure 1.

### Research instruments

*Quantitative study*

In order to assess the leaders’ knowledge and attitude regarding e-cigarettes, three parts of the questionnaire were used. The questionnaire, consisting of 32 items, was developed after a review of the literature^25,26^. The first part included demographics (3 items: age, sex, and faculty). The second part included an assessment of knowledge about e-cigarettes (16 items: relevant laws, substances, and risks). This section consisted of binary choice questions (yes or no). Each correct answer received 1 point, while each incorrect answer received 0 points. The total score of the knowledge section was 16. The third part included an assessment of attitudes toward e-cigarettes (13 items: acceptance, regulations, and harmfulness). The attitude part of the questionnaire comprised three response option: ‘agree’, ‘not sure’, and ‘disagree’. Positive questions received 3, 2, and 1 points, respectively, while negative questions received 1, 2, and 3 points. The total score of the attitude section was 39. The questionnaire’s item-objective congruence test resulted in a number ranging between 0.6 and 1.0, and the reliability of the questionnaire was acceptable (Cronbach’s α>0.7).

*Qualitative study*

In order to assess the situations of undergraduate students’ e-cigarette use and attitudes toward them, 18 undergraduate studentσ were asked questions using in-depth interview guidelines. Eight items of in-depth interview guidelines included the accessibility, vaping behavior, and incentivizing and disincentivizing features of e-cigarettes.

To evaluate the effect of the anti-e-cigarette campaign, 28 student leaders were requested to participate in a focus group. Ten items of focus-group discussion guides were used to ask the leaders questions regarding the outcomes of each campaign activity, the development of their communication skills, and the lessons learned from contributing to the campaign. The content validity of the in-depth interview guidelines was verified by three experts before use.

### Data analysis

For quantitative data analysis, the Statistical Package for the Social Sciences (SPSS), version 22.027, was used to carry out the statistical calculations. Descriptive statistics, such as frequency, percentage, mean, and standard deviation (SD), were employed to examine the participant characteristics. In order to determine whether a variable has a normal distribution, the Kolmogorov-Smirnov test was performed. To compare the average number pertaining to knowledge and attitude about e-cigarettes before and after the training program, the Wilcoxon signed rank test was employed, with a significance level of <0.05. Inductive thematic analysis of the content was used to analyze the data for qualitative data analysis. The first author transcribed the interview data verbatim from the digital recordings. The data were organized and coded using NVivo 1428, a qualitative software program. The first author initially coded data for content pertaining to the situations of undergraduate students’ e-cigarette use, attitudes about e-cigarette use, and the effect of the anti-e-cigarette campaign. With a primary focus on identifying prominent themes throughout the responses, descriptive codes based on patterns found in the data were gathered. These themes were discussed with the second author, revised, and validated by all team members.

## RESULTS

### The situations describing the use of e-cigarettes by undergraduate students and their attitudes toward their use

During the diagnosing stage, the situations of undergraduate students’ e-cigarette use and attitudes toward them were assessed with 18 undergraduate participants. The age of the participants was in the range of 18–21 years. Most of them were male (n=12; 66.7%). Regarding the use of e-cigarettes and their attitudes toward them, three themes were identified as follows.

*Access and context*

Most users believed that e-cigarettes were simple to purchase, even though Thailand forbids the sale of e-cigarettes in domestic shops. All users (n=9) access them from online shops:

*‘It is purchased online. There is no age check; therefore, it is not particularly difficult.’* (male, 19 years)

Many users claimed that they could access various vaping content on social media platforms. They also reported that online marketing strategies such as giveaways, discounts, or free trials encourage them to buy and consistently use:

*‘When you look at those online videos, these professionals can do amazing tricks.’* (male, 20 years)

The majority mentioned lending or borrowing e-cigarette equipment as well as group vaping with friends. They also emphasized the social connection component of their vaping by connecting the practice to certain social circumstances, such as hanging out with friends or going to events:

*‘I vape primarily for social reasons, fashion, and because it has a more festive vibe than smoking.’* (male, 18 years)

*Incentivizing features of e-cigarettes*

The majority of users claimed that e-cigarettes were easy to use and did not smell like conventional cigarettes:

*‘I find using the vape to be more convenient. Simply said, they didn’t make you smell.’* (female, 19 years)

They also mentioned that e-cigarettes were considered socially acceptable; e-cigarette use was acceptable when other people were around.

Most users also revealed that because of the tobacco-free smell, they could use e-cigarettes in locations where it was prohibited to do so, such as bedrooms, bathrooms, and some public places on campus, such as the back of the education buildings, as a result, the frequency of vaping increased:

*‘The novelty of being able to vape anywhere and how much simpler it was, were the attractions.’* (female, 20 years)

Most users also noted the variety of e-liquid flavors and the taste of e-cigarettes as factors encouraging their use of e-cigarettes:

*‘Yes, I believe that the option to customize the flavor is what causes people to choose e-cigarettes over traditional cigarettes.’* (male, 21 years)

Most users reported that e-cigarettes were safer than conventional cigarettes because the devices heat tobacco without burning. Additionally, they saw e-cigarettes as one of many methods for quitting smoking:

*‘I believe that using e-cigarettes could benefit people in quitting smoking.’* (male, 20 years)

*Disincentivizing features of e-cigarettes*

The negative health impacts were the disincentive aspect of using e-cigarettes, cited by some e-cigarette users. These were predominantly respiratory; the users stated that e-cigarettes had an impact on their throats, lungs, and ability to cough:

*‘I go for a short run and immediately begin to cough violently; it’s awful. However, I still vape.’* (male, 20 years)

For non-users, the vapor smell and health effects were their major reasons for not using e-cigarettes. Most non-users mentioned a bad odor:

*‘I don’t like it, and yes, it smelled unpleasant.’* (female, 19 years)

They also reported that they did not want to experience negative health reactions to e-cigarette use:

*‘I have never tried vaping, and I never will because it is bad for health.’* (male, 20 years)

Contrary to previous attempts at quitting, the majority of non-users mentioned that using an e-cigarette could be an alternative to smoking:

*‘I believe smokers who use e-cigarettes have only developed a new addiction and may not be able to quit smoking.’* (female, 20 years)

### The effectiveness of the intervention program in developing e-cigarette control leaders

During the planning action stage, the information on the diagnosing stage was used to develop an intervention program for developing e-cigarette control leaders. After the intervention program (taking action stage) was finished, the research team evaluated the program at the individual and group levels (evaluation action stage).

*Pre-test and post-test scores pertaining to knowledge and attitude*

In order to evaluate the effectiveness of indoor training activities in the program, individual-level changes in the leader’s knowledge and attitudes regarding e-cigarettes were assessed by 28 student leaders. All leaders completed the pre-test and post-test. The average age of the leaders was 20 years (SD=0.9). More than half of the leaders were female (60.7%) (Table 1). Half (50%) of the leaders were studying in the Faculty of Public Health. After evaluating the effectiveness of the indoor training activities, the results showed that the program could enhance knowledge and attitude regarding e-cigarettes. In the area of knowledge about e-cigarettes, the post-test mean score was greater than the pre-test mean score (mean=15.1, SD =0.7, range: 14–16 vs mean=13.6, SD=1.3, range: 9–16, p<0.001). Similarly, the post-test mean score for attitude toward e-cigarettes was greater than the pre-test mean score (mean= 36.3, SD=3.3, range: 27–39 vs mean=34.1, SD= 4.3, range: 23–39, p=0.001).

**Table 1:** Demographic characteristics of undergraduate student leaders of the University in Lampang Province, Thailand (N=28)

*Characteristics*	*n*	*%*
**Age** (years)		
18	1	3.6
19	7	25.0
20	11	39.3
21	9	32.1
**Sex**		
Male	11	39.3
Female	17	60.7
**Faculty/College**		
Faculty of Public Health	14	50.0
Faculty of Law	12	42.9
College of Interdisciplinary Studies	2	7.1

*The effect of the anti-e-cigarette campaign*

In order to evaluate the effectiveness of outdoor training activities of the program, group-level changes in the development of their skills in managing campaign activities were assessed. After the student leaders were invited to create an anti-e-cigarette campaign (outdoor training activities), they enjoyed the opportunity to learn and share ideas with others in groups and the creative freedom that came with making group presentations:

*‘We felt free to create our campaign. We think it was quite well-suited to undergraduate students.’* (male, 20 years)

The campaign included various activities, such as exhibitions, lectures and seminars, quizzes, e-posters, short films online, and printed posters. The leaders specifically set up online pages for this campaign. After the implementation of their campaign, the leaders were interviewed about the outcome, as follows.

The exhibition ‘Be smart, don’t start vaping’

In the beginning, the exhibition entitled ‘Be smart, don’t start vaping’ took place at the flea market on the campus for one day. The exhibit highlighted the harmfulness of e-cigarette use, and undergraduate student visitors completed numerous quizzes to discover how e-cigarettes impacted their health. An anti-vaping message delivered by peers of their age was likely to be effective. Although all the leaders had no prior experience in having conversations about e-cigarettes with peers, most felt confident about delivering their message:

*‘Many students were interested in our exhibition. When they asked us about the harmful effects of e-cigarettes, I could explain what I learned from the training.’* (female, 19 years)

Lecture and seminar

The tobacco treatment specialist was an invited guest lecturer on the topic of e-cigarettes. Twenty undergraduate students participated, and they were urged to use what they learned in the lecture to complete small-group projects and take part in exchanges about how to prevent e-cigarette initiation and promote early cessation among undergraduate students:

*‘They (students) suggested that the harmful effects should be more publicized and that regulations to discourage vaping in university should be stricter.’* (female, 20 years)

Online quizzes

The winning prizes of the online e-cigarette-quiz competitions were sent to the winners. Approximately 50 undergraduate students took part in the competition. The leaders reported that the quizzes supported student learning about e-cigarettes:

*‘A lot of students completed the quizzes. It might make it more enjoyable and assist students in learning about e-cigarettes.’* (male, 19 years)

E-posters

The leaders promoted the posters that contained advice on quitting e-cigarettes and the prevention of vaping on their online pages and the university clubs’ social media platforms. Despite the fact that over twenty posters were distributed, fewer students than anticipated actually shared. The leaders reported that it was challenging to engage students; their social media channels might not reach most undergraduate students:

*‘Since we just recently started our channels, many undergraduate students might not be aware of it. Even if we also post information on our university club’s page, it is insufficient to reach them.’* (female, 21 years)

Short film online

The anti-vaping short film was posted on their online page. This film aimed to increase awareness of the harmful effects of e-cigarette use, prevent e-cigarette initiation, and motivate e-cigarette users to quit. One month after the film was released, there were 30 views on the online page:

*‘Like the e-posters, it was hard to reach most undergraduate students and get them interested in our online information.’* (male, 20 years)

Printed posters

The printed posters were created from a poster design contest. The top three winning posters were selected to be displayed in many places on the campus. Ultimately, they succeeded in triggering thoughts about e-cigarettes among those who had not previously considered the possible consequences:

*‘After we stuck the posters on the board, many students first looked at it, and then they actually read it. They told me that the content in these posters could raise awareness of the harmful effects of e-cigarettes.’* (male, 19 years)

The leaders reported that all campaign activities were implemented as planned. This campaign was effective overall, according to what the leaders reported. Many undergraduate students joined the campaign activities and had conversations about e-cigarettes. Their knowledge increased and attitude improved regarding e-cigarettes, as proven by their quiz answers and informal interviews with the leaders. Moreover, there was evidence that three student users went to consult a nurse at the campus first-aid room on how to quit vaping, at the end of the campaign. However, e-cigarettes were not a priority issue for students. Many students still were not interested in the campaign activities:

*‘They [students] were not concerned that e-cigarette use was the problem issue on campus.’* (male, 20 years)

In order to increase the efficacy of the campaign well ahead, the leaders recommended disseminating online media more widely across a variety of online platforms, establishing a cooperative network with other organizations, and expanding the e-cigarette campaign beyond the campus.

After finishing the campaign, the leaders reported a greater-than-expected development in their soft skills and management skills for e-cigarette control:

*‘I think my management skills were developed through practical experience as a manager of the campaign projects. I can handle managerial responsibilities and promptly resolve issues when they happen.’* (female, 20 years)

Regarding soft skills, improvements in self-confidence, leadership, teamwork, communication, and social skills were among the benefits perceived. Many leaders stated how the program had given them the confidence they needed to discuss e-cigarettes with others in their age group:

*‘I think of myself as having improved. Working on this campaign increased my self-confidence with my educated friends on e-cigarettes.*’ (female, 19 years)

Despite being student leaders, they noted that conducting the campaign had improved their leadership abilities:

*‘Throughout our project, leadership was more apparent because we all understood that there were some tasks that needed to be finished.’* (male, 19 years)

## DISCUSSION

From the interviews that were held, an important finding was that the nationwide preventive actions being taken by Thailand against vaping appeared to be undermined by a new online and market arena. Young users in our study mentioned that e-cigarettes could be easily purchased online. The items were seen as being simple to purchase, like in countries where e-cigarette sales are less restricted^29,30^. This study suggests that authorities ought to be more stringent in prohibiting the sale of electronic cigarettes, especially via online marketplaces.

Young users in our survey identified social media as their main platform for e-cigarette use and their associated interaction rituals. Postings that promoted vaping positively were described as being generally accessible and quite popular^31,32^. Young people who have never used cigarettes or e-cigarettes are more susceptible to using them after being exposed to even low-intensity e-cigarette advertising^33^. Their vulnerability to e-cigarette promotion is a clear indicator of the need for stricter and consistent restrictions on modern international advertising channels like social media^34^.

Young users in our study perceived e-cigarettes as a social tool since they gave them the opportunity to connect with other peer users. These results were consistent with previous studies^29,35,36^, which reported that e-cigarettes provided social opportunities and group acceptance. In our study, incentivizing features of e-cigarettes included smell, social acceptability, more extensive use, taste/flavors, and safety, confirming findings from other studies^37-40^. For disincentivizing features, adverse health effects were mentioned by both users and non-users, confirming similar findings by others^39,41^.

As found from the studies^39,40,42^, young users in our study thought that using e-cigarettes is safer than conventional cigarettes. They also believed that e-cigarettes could help smokers to quit smoking. However, those attitudes needed to be corrected to avoid mistaken conceptions. Even though e-cigarettes did not contain tobacco, they still included nicotine along with a variety of other chemicals and additives. These substances are toxic and harmful^43^. Moreover, they could not be utilized as a therapeutic tool to help consumers quit or reduce their smoking^43,44^.

As found in previous studies^14,45^, the PAR used in this study was a useful instrument for involving young people in the development of public health initiatives and youth-driven activities and for encouraging transformational community change related to tobacco use. The anti-e-cigarette training program that included general details on the negative effects of vaping, as well as enacting a law on e-cigarette control, were suggested as being more effective^46^. Similar to the previous studies^47-49^, it was found from the results of our study that the knowledge and attitude regarding e-cigarettes of the student leaders were significantly improved by the training program over the short-term.

Peer leaders are a useful tool for running campus-based health programs. Intervention initiatives are run by individuals with central authority who encounter more resistance than peer leaders who are associated with them^50^. Peer leaders have the capacity to alter cultural practices, which can change the way in which people perceive tobacco products^21^. In addition to vaping-prevention interventions, the development of peer leaders in e-cigarette control in our study may have implications for other health-related issues, such as alcohol and drug use^51^.

In addition to enhancing knowledge and attitude regarding e-cigarettes, there is proof that the leaders gained other benefits from the intervention. The leaders in our study reported significant gains in self-confidence, leadership, working as a team, communication, and social and management skills for e-cigarette control. This result is in line with other PAR studies that demonstrate the value of practical experiences for students to develop authentic leadership skills^52^. This method of involvement in extracurricular activities used in this study was based on the ‘learning by doing’ principle, which provides opportunities for leaders to examine problems and find solutions by putting theoretical knowledge to work and investigating the underlying causes of a situation^53^.

The use of social media to inform young people about e-cigarettes proved successful^54^. Student leaders in our study also used social-media formats, including visuals and quizzes in e-cigarette education. The message designs for those two formats were developed using a ‘mix of attributes’ theory^55^ that had been found to increase the intended message reactions, such as thinking. As in several other studies^54,56^, the social-media messages in our study could support undergraduate students learning about e-cigarettes and improve attitudes about the possible harm involved in the use of e-cigarettes. However, the leaders in our study reported the low reach of their social-media messages. One possible explanation for this could be that their social media platforms were unknown, and a small number of students knew about them. Therefore, a variety of widespread online platforms, such as the university website and celebrity websites, should be used for further campaigns. In addition, low perceptions of e-cigarettes as harmful among undergraduate students might be another reason for low engagement with online media^57^. The larger campaign on the campus to raise awareness about the dangers of e-cigarette use should be targeted at the students to increase their engagement in future campaigns.

### Strengths and limitations

The strengths of this study are that participants provided detailed insights into the issue of e-cigarette use among undergraduate students and demonstrated the value of using a PAR approach to develop the intervention program to develop e-cigarette control leaders in the university setting. This level of disclosure was made possible by the anonymity of the procedure. The study had unavoidable limitations. Firstly, self-report data were used to measure all outcome variables. Because of social desirability, this method of measurement is subject to response bias. Secondly, this study had a small sample size, which might have hindered generalizability. Thirdly, this study lacked a control group. Thus, potential confounding factors might interfere with and undermine the validity of the findings. Fourthly, using lottery methods to select student leaders who applied for the training program could lead to sample selection bias.

## CONCLUSIONS

The results of our study show that there were concerning situations surrounding the use of e-cigarettes by undergraduate students and their attitudes toward their use. The users viewed e-cigarettes as a more accessible social tool than conventional cigarettes, and they were also safer, more attractive, and more socially acceptable. The vapor smell and health effects were the primary reasons for not using them. The intervention program aimed at developing leaders in e-cigarette control has produced positive results. This intervention could significantly enhance the leaders’ knowledge and attitude regarding e-cigarettes. Following their anti-e-cigarette campaign, the soft skills and managerial abilities of the leaders in e-cigarette control improved. This intervention is recommended as a means of discouraging and preventing undergraduate students from using e-cigarettes.

## Data Availability

The data that support the findings of this study are available from the corresponding author, upon reasonable request.
